# Silicone as a smart solution for simulating soft tissue—an iterative approach to developing a high-fidelity sustainable training model for laparoscopic appendectomy

**DOI:** 10.3389/fsurg.2024.1483629

**Published:** 2024-11-21

**Authors:** Adam F. Roche, Gabrielle Diebold, Niamh McCawley, William P. Duggan, Andrea J. Doyle, Tim Lawler, Caoimhin O’Conghaile, Claire M. Condron

**Affiliations:** ^1^ RCSI SIM Centre for Simulation Education and Research, RCSI University of Medicine and Health Sciences, Dublin, Ireland; ^2^ School of Medicine, RCSI University of Medicine and Health Sciences, Dublin, Ireland; ^3^ Department of General Surgery, Beaumont Hospital, Dublin, Ireland

**Keywords:** simulation, patient safety, laparoscopy, appendectomy, surgery

## Abstract

**Background:**

Laparoscopic appendectomy (LA) is an effective treatment for the surgical care of appendicitis, with this minimally invasive approach allowing patients to typically spend less time in hospital and promptly return to normal life activities. Residents can acquire the competence and confidence needed in a safe learning environment prior to real patient encounters through simulation-based learning of these techniques. We propose a low cost, sustainable, high fidelity simulation-based training model for LA to compliment regular resident practice of these skills.

**Methods:**

A team dedicated to developing this surgical simulation training model was established, equipped with the clinical knowledge and model engineering expertise. We used concepts of design-based research (DBR) to iteratively develop this model at key intervals. Our LA training model underwent four stages of model development prior to unified stakeholder consensus that this model was deemed effective and suitable for integration into formative surgical simulation curricula.

**Results:**

This model simulates most of the key anatomical structures associated with performing an LA. In order to provide high fidelity haptic feedback, attempts were made to mimic the tensile properties of real tissue using different concentrations of silicone. The model can be utilized with laparoscopic box trainers of various sizes due to its scalability. It cost €9.67 to create, and single use appendix components cost €1.22 to build thereafter.

**Conclusions:**

Surgical residents can benefit from the platform that simulation-based education offers to develop the psychomotor skills necessary to perform LA in a safe learning environment. We describe a model for LA, which allows learners to develop their skill proficiency in this area under expert supervision.

## Introduction

Laparoscopic appendectomy (LA) is the gold standard for the surgical care of appendicitis, with this minimally invasive approach allowing patients to typically spend less time in hospital and promptly return to normal life activities (1). It is deemed an ideal training opportunity for young surgeons owing to the high volume of patients who present requiring this surgery (2), most cases are deemed technically straightforward, and it is not associated with high morbidity or mortality rates (3).

Despite all of this, key opinion leaders give this procedure high priority for evaluating resident competency in a simulation-based setting (4). Standardized procedural checklists within an organised training environment, involving both trainers and residents, have been shown to be useful training paradigms (5). Alongside this process, simulation-training models enable learners to rehearse critical procedural steps and develop the necessary psychomotor skills in a safe learning environment.

Although cost-effective biological model equivalents are useful, there are limitations on their biological application in laboratories that aren't licensed to use such specimens (6). In contrast, virtual reality simulators (7), and to a lesser extent, single-use commercially made synthetic training models may carry a significant financial burden on the delivery of these training programmes. Silicone is a moldable material that can be used to effectively replicate soft tissue. It is available in a range of shore hardness levels, from soft to hard. There is a growing need to create simulation-training models of low cost and high-fidelity, to enable construction and subsequent use in low resource settings (8).

We describe a high fidelity, low cost, sustainable, simulation-based training model for LA that can be replicated at large scale, using different representations of variegated silicone as a proof of concept.

## Method

Concepts from design-based research (DBR) were used to guide the evolutionary design and model development process over a period of 6 months (November 2023—May 2024) (9). Engineering methods that constitute evaluative and incremental improvement elements are becoming more prevalent in simulation-based education; with the aim of implementing continuous improvement cycles to effectively address learner needs (10). The main goal of this LA model development was to create a model that could be used for experiential learning (11), specifically for large cohorts of surgical residents to engage in deliberate practice (12).

**Figure 1:**
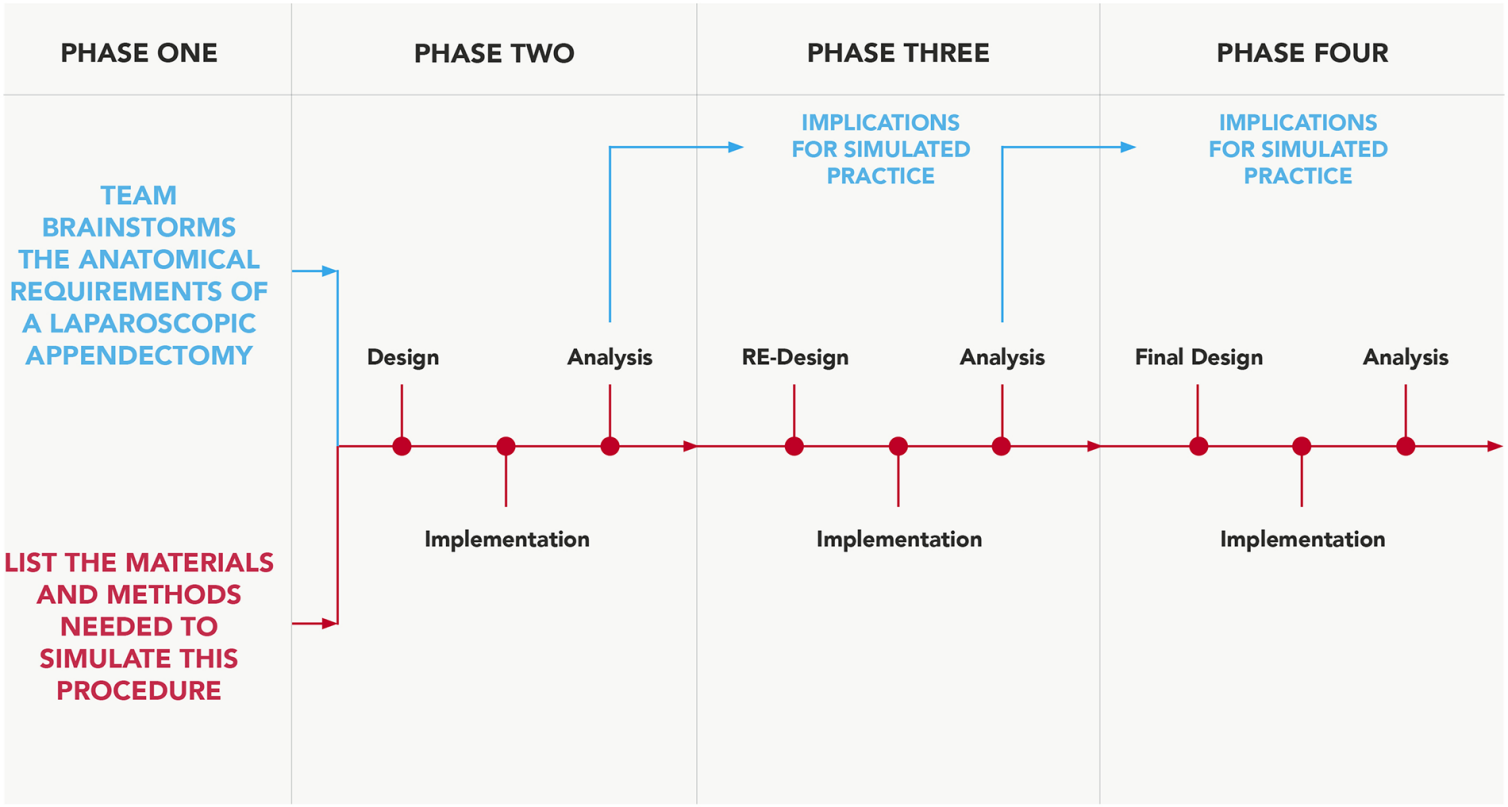
Design-based approach. Process adapted and modified for simulation-model development, from Fraefel (13).

As a framework template, a predetermined procedural checklist was also used to ensure the model replicated the majority of the previously specified procedural steps (5). A team dedicated to developing this surgical simulation-training model was established, equipped with the clinical knowledge and model engineering expertise. This comprised three simulation-based researchers (AR, GD, COC), a consultant general surgeon (NMcC), a junior general surgical registrar (WD) and a simulation-based educationalist (CC). Figure 1 outlines the critical steps of this process.

Phase one design was a singularly occurring event, led by AR, GD, COC and CC, who have extensive experience utilising cadaveric visceral tissue for simulation-based training and have considerable expertise in replicating surgical anatomy with synthetic materials (14–16). This incorporated an anatomical review of appendix anatomy (5, 17), subsequent brainstorming and documentation around potential materials to use for physical model creation. Arising from a review of the literature and brainstorming with colleagues, it was agreed to utilise various shore hardness and colorants of silicone to make the model in its entirety for phase two testing, as previous research has reported success in utilising silicone to mimic deep tissue biomechanics (18). The fact that silicone does not adhere well to most other synthetic materials besides itself was also a reason for this selection; the use of a single material type would enable efficacious model composition. It was decided not to obtain clinical input until phase two onwards in order to avoid feedback saturation.

The remaining testing and feedback phases comprised task execution and analysis, with surgical input from NMcC and WD at separate intervals, in order for the remaining team to evaluate and collate model usability feedback from both surgeon attempts in isolation. Both surgeons have carried out multiple laparoscopic appendectomies as the primary operator, NMcC (>800), WD (≥) 50. An objective approach was taken to procedural execution for task assessment, utilising some of the steps outlined in a previous study (5), to also include clipping of the appendicular artery. Reflection and detailed analysis on the models usability followed task assessment from both user attempts combined.

The author team could not find any validated rubrics in the literature, which seek to evaluate the usability of simulation models; therefore, we devised a traffic light system evaluation method, which records and maps the progression of qualitative user feedback from all iterative feedback phases (Table 1). This evaluation method focused on three main areas: (1) tensile qualities, (2) tissue discernibility and (3) anatomical scale and location. Both surgeons would need to be in unanimous agreement that functional fidelity (19) has been achieved for each of the seven components before green light status can be achieved.

One simulation-based researcher (AR) led the documentation process, and convened with the remaining research team to discuss user feedback and map the terrain for further model development. The majority of the surgical input for each component's improvement was based largely around anatomy that was either too thin or thick, incorrect colour, improper fixation or tissue adjoining to the incorrect anatomical location. Our LA training model underwent four stages of model development prior to unified stakeholder consensus that this model was deemed effective and suitable for integration into formative surgical simulation curricula, thus all components obtaining green traffic light status. Additional surgical evaluation occurred during phase three in order to somewhat expedite the model development process.

**Table 1:** Overview of development timeline for each component of the simulation-model.

Phase	01		02				03				04		
	Week (✪ = Deliverable, *◆* = Milestone)												
Tissue component	02	04	06	08	10	12	14	16	18	20	22	24	26
Retroperitoneal base			✪	◆									
Usability feedback				✓									
Greater omentum			✪				◆						
Usability feedback			T				✓						
Large bowel			✪				✪				✪	◆	
Usability feedback		T, D, S				T, S					S	✓	
Small bowel			✪				✪				✪	◆	
Usability feedback		T, D, S					T,S				S	✓	
Appendix			✪				✪		✪	◆			
Usability feedback		T, D, S				T, D			T	✓			
Mesentery			✪				✪	◆					
Usability feedback		T, S					T	✓					
Vasculature			✪				✪		✪	◆			
Usability feedback		D, S					S		S	✓			
Overall model			✪				✪				✪		◆

Deliverable outlines the intervals during which the revised model was evaluated. Milestone denotes the intervals when both surgeons agreed that the relevant model component was deemed effective. Areas for improvement arising from combined clinical feedback indicators: T, tensile qualities; D, tissue discernibility and S, scale and anatomical location.

## Results

**Figure 2:**
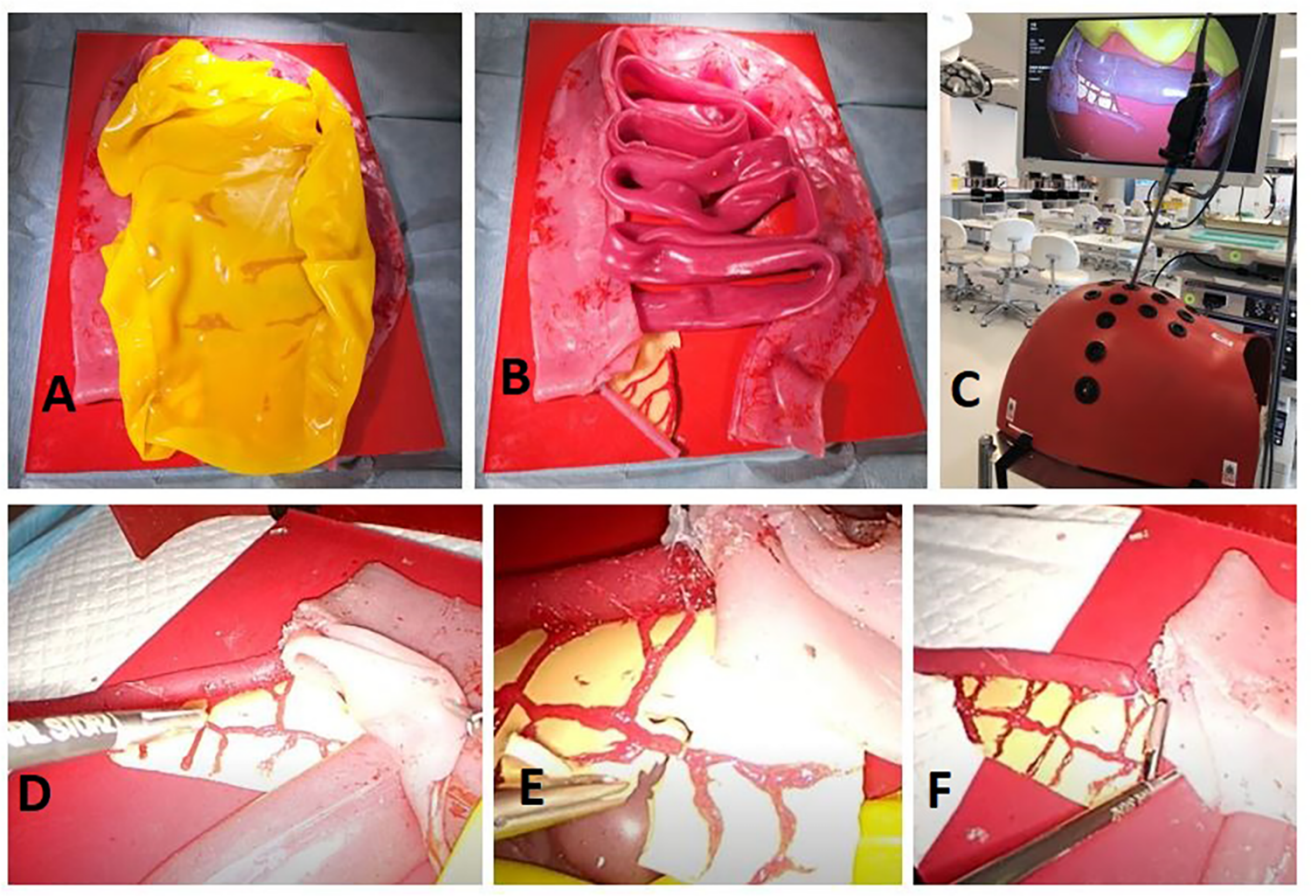
Entire model fixed to retroperitoneal base covered in a sheet of greater omentum **(A)** appendix and mesoappendix visibly connected to caecum and ileum **(B)** model view from inside laparoscopic box trainer **(C)** retraction of caecum to isolate appendix **(D)** division of mesoappendix and clipping of appendicular artery **(E)** endoloop placement prior to division of appendix **(F)**.

Our simulation training-model effectively allows residents to execute most of the critical steps associated with performing an LA. The visceral anatomy sits on top of a hard red silicone retroperitoneal base. The small and large intestines are adhered to different areas of the base in order to prevent full lift off throughout practice. The entire model is inserted into a laparoscopic box trainer; since the trainer has already established abdominal access, it is not possible to practice abdominal entry through pneumoperitoneum execution. The model design approach is scalable to accommodate different sized box trainers (Figure 2). Appendix 1 contains a video of one author (NMcC) performing procedural tasks on this model; Appendix 2 provides step-by-step instructions on how to build this model.

Of the intestinal anatomy, only the caecum and ileum are physically required for task execution in this model. We decided to recreate the entire small and large bowel tracts since they are reusable, inexpensive to construct, and gives residents an opportunity to immerse themselves in the task as a whole through representation of additional intestinal anatomy. Representation of the greater momentum adds another step to the procedural process, as residents must retract this prior to task progression.

Mesoappendix and surrounding vasculature are represented with anatomical accuracy in this model, in terms of discernibility, anatomical placement and tensile qualities. This allows residents to effectively divide the mesentery, clip the appendicular artery and continue with the appendix division. One limitation of the model, though, is that, unlike in reality, where surgeons usually separate the mesentery using diathermy, the mesentery is not reactable with energy devices. However, it does allow for practice of careful tissue handling with a laparoscopic scissors.

Crucially, the silicone used to make the appendix is more durable, in order to give a realistic tensile feel, and without being too soft that endoloop tightening would cause an ill-timed division. This was given a lot of consideration throughout the model development process. The appendix is attached to the caecum using a button fastener, meaning the only single use aspect of this model are the appendix components, which are time and cost effective to replace. This model allows for different representations of the task, as the appendix can be positioned retrocaecul, retroileal and in the normal lying free position. A single model in its entirety costs €9.67 to create, and single use appendix cost €1.22 to build thereafter. This, however, does not take into account hidden costs that are difficult to quantify, such as the time technicians require to assemble the model and laparoscopic equipment, such as instruments, box trainer, light source, camera and monitor.

## Discussion

Limited information is available in the literature regarding the description of low-cost synthetic simulation-training models for LA. Due to the wide range of conceivable silicone stiffness properties, every anatomical region in our model is fabricated with tensile qualities that resemble real tissue as closely as possible. Using iterative qualitative feedback from experts at critical phases in the model development process, we were able to produce a high-fidelity, low-cost training model for LA that serves as a useful training tool for junior surgeons.

The European Association of Endoscopic Surgery (EAES) recommend that surgical residents need to perform 20 surgeries during the learning curve phase, in order to be able to perform these surgeries independently (20). Through deliberate practice (12), simulation-training utilising this LA simulation model can enhance performance automation, which supports transfer of skills to the operating room (OR) environment. Enhanced task performance through simulation reduces cognitive load in the OR, enabling residents to have greater use of nonrecurrent skills such as decision-making and problem-solving (21). As a result, residents are better equipped to deal with uncertainty and cope with other issues as they arise throughout operating.

Simulation-based education can be costly. Our LA simulation model is economically manufactured, but as previously stated, it cannot be used in isolation; additional equipment and consumables are required for complete laparoscopic practice. As seen in Figure 2, laparoscopic equipment of a clinical standard is prohibitively more expensive, rendering it unaffordable and unattainable in many institutions and jurisdictions. However, a host of affordable laparoscopic setups are available for home creation, as previously described (22, 23). Another study describes a simple laparoscopic setup that uses supplies that can be purchased at a hardware store (24), the only expensive aspect being instrumentation. However, we were able to obtain a catalogue of cost-effective laparoscopic instruments ranging from €22.65, from internet-based shops, which can mitigate costs associated with purchasing costly instruments. By combining our LA model with a cost effective and impactful laparoscopic simulation training setup, residents in the western world and in low resource settings (25) can engage in affordable and meaningful practice of these skills.

Our model has some limitations. In the event of vascular perforation, the vasculature in our model does not bleed. Future designs of the model should address this issue. As previously described, our model is not reactable with energy devices. While some synthetic materials, such as hydrogel, do function in this way, their cost is far higher. Further research should focus on utilising established methods (26, 27) to collate validity evidence to evaluate the model further in order to quantifiably determine whether the simulation-model performs as intended. Previous studies have showed positive correlational outcomes between iterative model design and collating sufficient validation metrics to deem a model effective (13, 28).

## Conclusion

Simulation-based education provides a useful platform for surgical residents to develop psychomotor skills in a safe learning environment. We describe a model for LA, which allows learners to develop their skill proficiency in this area under expert supervision.

## Data Availability

The original contributions presented in the study are included in the article/Supplementary Material, further inquiries can be directed to the corresponding author.

## Appendix

### Appendix 1 Video recording of expert performance of laparoscopic appendectomy using this model

Expert video recorded performance https://www.youtube.com/watch?v=IKgowAlIDGs.

## Appendix 2 Step-wise instructions on how to build the model

**Table:**

Feature	Components	Amount (e.g., weight/percentage in mixture)	Procedure
Appendix	SmoothOn^TM^ EcoFlex Silicone 00-30 (A)	10 ml	Place the modelling clay on the table and stand the straw upright in it. Ensure it will not fall over. Mix materials together in the container. Open the syringe, fill with the mix and then slowly fill the tube/drinking straw. Allow to set for at least 6 h. Pull out and cut to 100 mm with scissors. Attach male button to one end with SmoothOn^TM^ SilPoxy and allow to set for at least 6 h
	SmoothOn^TM^ EcoFlex Silicone 00-30 (B)	10 ml	
	SmoothOn^TM^ THI-VEX (thickener)	10 ml	
	Red flocking powder (any brand)	5 ml	
	SmoothOn^TM^ Pigment, Red	Pinch	
	8 mm interal diameter tube, e.g., drinking straw	1 drop	
	SmoothOn^TM^ SilPoxy	Tube ×1	
	Modelling clay	Golf ball size	
	20 ml syringe	×1	
	Male button	×1	
	500 ml container	×1	
Mesentery	SmoothOn^TM^ EcoFlex Silicone 00-10 (A)	100 ml	Mix parts and allow to set on a flat contained surface for at least 6 h
	SmoothOn^TM^ EcoFlex Silicone 00-10 (B)	100 ml	
	SmoothOn^TM^ Silicone Thinner	100 ml	
	SmoothOn^TM^ Pigment Light Flesh	1 drop	
	SmoothOn^TM^ Pigment Yellow	1 drop	
	500 ml container	×1	
Appendicular artery	SmoothOn^TM^ SilPoxy	10 ml	Mix and place into a 10 ml syringe. Press out the material onto the set mesentery as shown in the image. Remove and safely dispose of the needle and attach the empty cannula to the syringe to extrude finer vessels. Allow to set for at least 6 h
	Syringe 10 ml	×1	
	SmoothOn^TM^ Pigment, Red	0.5 drop	
	Cannula 14G	×1	
Greater omentum	SmoothOn^TM^ EcoFlex Silicone 00-10 (A)	300 ml	Combine and mix the materials in the container. Allow to set on a flat contained surface for at least 6 h
	SmoothOn^TM^ EcoFlex Silicone 00-10 (B)	300 ml	
	SmoothOn^TM^ Pigment Yellow	3 ml	
	Syringe 5 ml	×1	
	Container 1 l	×1	
Small Intestine	SmoothOn^TM^ EcoFlex Silicone 00-10 (A)	300 ml	Combine and mix in the container. Mix parts and allow to set on a flat contained surface for at least 6 h. Cut shape 600 × 300 mm. Roll into a tube and seal closed with SmoothOn^TM^ SilPoxy adhesive and allow to set for 6 h
	SmoothOn^TM^ EcoFlex Silicone 00-10 (B)	300 ml	
	SmoothOn^TM^ Pigment Red	3 ml	
Cecum and ascending colon	SmoothOn^TM^ EcoFlex Silicone 00-10 (A)	250 ml	Mix parts and allow to set on a flat contained surface for at least 6 h. When set, detach from the table and place the foam on the silicone and wrap loosely. Seal closed with SmoothOn^TM^ SilPoxy adhesive. Allow to set for 6 h. Attach female button to the cecum with SmoothOn^TM^ SilPoxy adhesive and allow to set for 6 h
	SmoothOn^TM^ EcoFlex Silicone 00-10 (B)	250 ml	
	SmoothOn^TM^ Pigment Red	2 ml	
	Female button	×1	
	SmoothOn^TM^ SilPoxy	10 ml	
	60 mm diameter foam	300 mm (length)	
Base mat	SmoothOn Mold Max40 (A)	910 ml	Using separate syringes to draw each material, measure the relative amounts. Combine and mix in the container. Place into the tray and allow to set untouched for a minimum of 24 h
	SmoothOn Mold Max 40 (B)	90 ml	
	SmoothOn^TM^ Pigment Red	5 ml	
	Syringe 500 ml	1	
	Syringe 100 ml	1	
	Syringe 5 ml	1	
	2l container	1	
	Tray approx	600 × 300 × 30mm	

Model Assembly Instructions:

•  Use incontinence sheets and personal protective equipment (plastic apron and gloves) before making the above models and during assembly to protect workspaces and clothes.

• Use specifically designated syringes of appropriate volume to draw exact quantities.

• Remove the base mat from its container and place on a flat surface in portrait orientation.

•  Place the caecum and ascending colon on the left side of the mat.

• Place the small intestine above the appendix and use SmoothOn^TM^ SilPoxy to adhese it to the caecum and ascending colon, shown below as the terminal ileum.

• Allow to set for at least 6 h.

• Cut the mesentery to shape, see image below.

• Place the appendix on the mesentery and use SmoothOn^TM^ SilPoxy to adhese.

• Allow to set for at least 6 h.

• Attach the male button of the appendix model to the female button of the cecum.

•  Place the greater omentum over the model.

• If required, more appendix models can be made by repeating the above steps to replace the mesentery, appendix and appendicular vasculature.
